# Difficulties in everyday life: Young persons with attention-deficit/hyperactivity disorder and autism spectrum disorders perspectives. A chat-log analysis

**DOI:** 10.3402/qhw.v9.23376

**Published:** 2014-05-28

**Authors:** Britt H Ahlström, Elisabet Wentz

**Affiliations:** 1 Department of Nursing, Health and Culture, University West, Trollhättan, Sweden; 2 The Vårdal Institute, Swedish Institute for Health Sciences, Lund, Sweden; 3 Gillberg Neuropsychiatry Centre, Institute of Neuroscience and Physiology, Sahlgrenska Academy, University of Gothenburg, Göteborg, Sweden

**Keywords:** ADHD, autism, coaching, content analysis, everyday life, Internet-based support, vulnerability, young persons

## Abstract

This study focuses on the everyday life of young persons with attention-deficit/hyperactivity disorder (ADHD) and autism spectrum disorder (ASD). There are follow-up studies describing ADHD, and ASD in adults, and residual impairments that affect life. Few qualitative studies have been conducted on the subject of their experiences of everyday life, and even fewer are from young persons’ perspectives. This study's aim was to describe how young persons with ADHD and ASD function and how they manage their everyday life based on analyses of Internet-based chat logs. Twelve young persons (7 males and 5 females aged 15–26) diagnosed with ADHD and ASD were included consecutively and offered 8 weeks of Internet-based Support and Coaching (IBSC). Data were collected from 12 chat logs (445 pages of text) produced interactively by the participants and the coaches. Qualitative content analysis was applied. The text was coded and sorted into subthemes and further interpreted into themes. The findings revealed two themes: “fighting against an everyday life lived in vulnerability” with the following subthemes: “difficult things,” “stress and rest,” and “when feelings and thoughts are a concern”; and the theme “struggling to find a life of one's own” with the following subthemes: “decide and carry out,” “making life choices,” and “taking care of oneself.” Dealing with the problematic situations that everyday encompasses requires personal strength and a desire to find adequate solutions, as well as to discover a role in society. This study, into the provision of support and coaching over the Internet, led to more in-depth knowledge about these young persons’ everyday lives and revealed their ability to use IBSC to express the complexity of everyday life for young persons with ADHD and ASD. The implications of the findings are that using online coaching makes available new opportunities for healthcare professionals to acknowledge these young persons’ problems.

This qualitative study focuses on the everyday life of young persons with attention-deficit/hyperactivity disorder (ADHD) and autism spectrum disorder (ASD). Until recently, ADHD and ASD research focused on children and adolescents. ASD is known to persist into adulthood, while ADHD is thought to dissipate in adolescence or adulthood. However, follow-up studies describe ADHD (Faraone & Biederman, 2005; Kessler et al., 2006; Rasmussen & Gillberg, 2000) and ASD (Billstedt, Gillberg, & Gillberg, 2005; Cederlund, Hagberg, Billstedt, Gillberg, & Gillberg, 2008) in adults with residual impairments that are life-affecting. In the US replication study of the National Comorbidity Survey (NCS), the estimated prevalence of adult ADHD was 4.4%, but only 1 in 10 received treatment for the disorder (Kessler et al., 2006). The prevalence of autism, calculated from 34 autism studies, was estimated at 13 persons per 10,000 and Asperger's syndrome (AS) at 3 per 10,000 (Fombonne, 2005). However, in a restricted area in Sweden, the minimum number of registered persons screened with ASD was 20.5 per 10,000 (Gillberg, Cederlund, Lamberg, & Zeijlon, 2006). Core symptoms of ADHD include inattention, hyperactivity, and impulsivity. ASD is characterized by social communication problems and stereotyped or repetitive behaviors. However, there is considerable correspondence regarding these two neuropsychiatric disabilities; 30% of adults with ASD have a co-existent ADHD (Hallerbäck, Lunegård, & Gilberg, 2012) and among adults with ADHD 30% also meet the requirements for an ASD diagnosis (Stahlberg, Soderstrom, Rastam, & Gillberg, 2004). Both ADHD and ASD have problems with executive functions (Barkley & Murphy, 2012; Nydén et al., 2010). Adults with ADHD, ASD, or both exhibit temperamental and personality traits that resemble personality disorders, indicating an increased risk of psychiatric co-morbidity (Ankarsäter et al., 2006). This co-morbidity almost certainly leads to a more complicated everyday life. A previous study compared the conditions of everyday life in young men with AS and normal intelligence with a group of adult men with autism and co-morbid intellectual disability (Cederlund et al., 2008). Conditions proved to be worse than expected for both groups and worst for the group of men with autism. Lack of education, work, activities, and friends resulted in a limited life. Both the AS and autism groups were dependent on parental support, regardless of whether or not they were living independently.

One essential part of a young person's everyday life, including those with ADHD and ASD, is school. In a qualitative study, college students with ADHD described factors that hindered and helped them to study in each of the global themes “gaining insight about ADHD,” “managing life,” and “utilizing sources of support” (Meaux, Green, & Broussard, 2009). Another part of a young person's everyday life is employment. Work has positive effects on global functioning in young people with mental illness, including individuals with neuropsychiatric disorders (NPD) (Nygren, Markström, Svensson, Hansson, & Sandlund, 2011). However, it is hard for young persons with ADHD to get a job (Asherson et al., 2012; Kessler et al., 2006), and it is even harder for those with ASD (Billstedt et al., 2005; Cederlund et al., 2008). Friends are important for young persons, and those with ASD have few friends (Cederlund et al., 2008). Whether or not a young person with ADHD is likely to be accepted by their peers depends on the age and gender of the friends and their perception of the ADHD sufferer's own responsibility for their condition (Swords, Heary, & Hennessy, 2011).

Studies have also been carried out concerning aspects of everyday life other than education and employment in adolescents with ADHD and ASD. Electronic diaries were used to investigate everyday life in the form of online self-reports. Adolescents (aged 14) with high scores for symptoms of ADHD spent more time with friends than with their families, preferred social activities to homework, reported more negative moods, and were less alert than those who had low scores for ADHD symptoms (Whalen, Jamner, Henker, Delfino, & Lozano, 2002). In a literature review of everyday problem solving (Channon, 2004), the author concludes that the complex procedures involved in performing everyday tasks are probably dependent on multi-cognitive and emotional solutions. This complicates both the understanding of how persons with Tourette's syndrome and AS, for instance, function and how they should be supported in everyday life. The processes involved in the performance of theory of mind tasks (eye tasks, strange stories, and stories from everyday life) in adolescents with high functioning autism or AS have been investigated (Kaland, Callesen, Mølle-Nilsen, Mortensen, & Smith, 2008) and show that their performance was poorer than that of a control group. The results indicate that adolescents with ASD have difficulties understanding and reacting adequately to events in everyday life.

Few qualitative studies have been conducted on the subject of experiences of everyday life of young persons with ADHD and ASD, and even fewer have a young person's perspective. Consequently, we lack knowledge about young persons' with ADHD and ASD perspectives on their experiences of everyday life.

## Aim

The aim of this study was to describe how young persons with ADHD and ASD function and how they manage their everyday life based on analyses of Internet-based chat logs.

## Method

A qualitative methodology was used in order to explore young persons’ experiences of everyday life (Patton, 2002). This study has been reported in two papers because of the large amount of data.

### Settings and procedures

This study took place in the south of Sweden from 2008 to 2010, and is part of an intervention project, NP Young Coaching, investigating whether Internet-based support and coaching (IBSC) can benefit the health and quality of life of adolescents and young adults with ADHD and ASD. In the present study, “young persons” are defined as adolescents and young adults aged 15–26.

#### Internet-based support and coaching

The model for IBSC was developed by the second author. A computer program, Salut Chat, was developed for young persons with NPD by EW in collaboration with the Swiss company Net Union. IBSC is meant to be a home-based and interactive intervention. The model includes 8 weeks of IBSC, comprising twice-weekly booked Internet-based chat sessions with a coach, each intended to last between 30 and 60 minutes. The model also includes face-to-face meetings, initially before the chat period followed by two meetings during the 8-week IBCS period. The coaches in the pilot study, three female psychologists and one female educational therapist, were all very experienced in meeting children, adolescents and young adults with NPD. The young persons had access to the chat program via the Internet, and the chat sessions usually took place in the participants’ homes at times convenient for them. The coaches chatted with the participants from their work places or homes (Wentz, Nydén, & Krevers, 2012).

### Participants

Young persons aged between 15 and 26 years, with ADHD, and ASD, diagnosed according to the Diagnostic and Statistical Manual of Mental Disorders (DSM-IV; American Psychiatric Association, 1994) were invited to participate. They were asked if they were in need of support and coaching in everyday life. The young person had to have access to a computer with an Internet connection at home. Young persons with a co-morbid current psychosis, alcohol or substance abuse, major depression (if it was too severe and therefore an obstacle to participating in the intervention), severe dyslexia, intellectual disability, or were currently involved in criminal activities were excluded.

Twelve young persons were consecutively included; seven males and five females, aged between 15 and 26 years (median 17 years, mean age 19,6 years). Ten participants had a diagnosis of ADHD of whom six had ADHD in combination with ASD or affective disorders. Six participants had some form of ASD; two had ASD alone and four in combination with ADHD. Two were attending elementary school, five upper secondary school, and one was at university. Four had finished school and were unemployed; two did not complete their participation in the IBSC.

### Data collection

Data were collected from the chat logs produced interactively between the participant and the coach. In total, the 12 chat logs comprised 445 pages of text. The chat-log data were rendered anonymous. The whole of the texts emanating from the chat sessions constituted the data for the qualitative analysis.

### Analysis

Qualitative content analysis is a well-known method for descriptive analysis of text, and is suitable for use with larger bodies of text (Krippendorff, 2004). It is performed step by step in order to describe patterns and themes (Patton, 2002). In content analysis, the text is divided into units selected in accordance with the research question. Each unit is then labeled with a code describing its content. Finally, the text is reduced and rewritten in the form of subthemes and themes derived from the preceding steps (Krippendorff, 2004).

The first author started the analysis by reading the whole body of the text in order to gain an overview of the data and simultaneously divided it into meaning units, i.e., parts of the text comprising the same content. This analytical processing yielded preliminary themes which guided the subsequent coding process carried out using the analysis program NVivo9, which was used as a support function to organise data during the analysis process. All the meaning units were labeled with codes which were compared regarding differences and similarities, and thereafter sorted into subthemes and interpreted into themes. This process meant that the subthemes and themes were carefully reflected on resulting in the subthemes being condensed and merged and the preliminary themes were reassessed to produce the final themes. All data from the chat logs were analysed. As the data are so extensive, the analysis presented in the current study comprises only a part of the findings. Themes with content focusing on everyday life were grouped together for the present manuscript. No participants were excluded and the results emerged from analysis of all chat logs.

### Ethical considerations

This study adhered to the guidelines set out in the World Medical Association Declaration of Helsinki (2008) and was approved by the Regional Ethical Review Board in Gothenburg (GU 013-08).

## Findings

The findings comprise two themes: “fighting against an everyday life lived in vulnerability” with the following subthemes: “difficult things,” “stress and rest,” and “when feelings and thoughts are a concern”; and the second theme “struggling to find a life of one's own” with the following subthemes: “decide and carry out,” “making life choices,” and “taking care of oneself.” An overview of subthemes and themes is given in Table I.

**Table I:** An overview of subthemes and themes.

Subthemes	Themes
Difficult things Stress and rest When feelings and thoughts are a concern	Fighting against an everyday life lived in vulnerability
Decide and carry out Making life choices Taking care of oneself	Struggling to find a life of one's own

### Fighting against an everyday life lived in vulnerability

#### Difficult things

In everyday life, the young persons were burdened by difficult things that were challenging and made them vulnerable. The young persons described frustration at being treated unfairly, e.g., not having received the same benefits from their parents as younger siblings had, and being regarded as mature and capable of providing for themselves. In their own eyes, this happened too early. Also receiving too small a weekly allowance, significantly lower than that recommended for their age, was a problem when negotiations with parents failed.

Gabriel: I want a little more money each week, maybe 5–10 kronor more.

Coach: What do you need the money for?

Gabriel: Because I want to be able to save a little every week.

Coach: That is a good idea! You say 5–10 kronor more, what do you get?

Gabriel: 20 kronor

Gabriel: And a bag of potato crisps costs 21 kronor

Coach: You mean 20 kronor a week?

Gabriel: Yes

Other examples of being treated unfairly were when the young persons described how they were undeservedly accused of being the cause of failure in the family when plans had to be postponed, of behaving egoistically, and of causing the parents to be in a bad mood. There were examples of parents sometimes speaking harshly to the young persons and of them being teased by siblings and friends. These experiences were described as being difficult to handle, and were a further source of sorrow and frustration.

Difficult offensive situations in school were described arising when schoolmates made fun of the young persons. The teachers seemed unable to prevent this behavior which approached harassment. In some cases, the teachers were described as the offenders, making the situation even harder to endure. Feeling offended by their actions resulted in a loss of respect for the teacher, and being laughed at by peers resulted in anxiety. Being let down by friends in favor of other peers was described as devastating as it was a source of misunderstandings that led to conflicts. The experience of being rejected by a parent in a tense family situation was perceived as resulting from incomprehension on the part of the stressed parent. There were also descriptions of experiences of being physically punished by a parent. In interpreting such difficult situations, the young persons used empathy to try to understand the parent's behavior. The young persons felt trapped in the middle of, and sometimes the target for, family conflicts, and not understanding what was going on.

Frida: No, not rowdy, but I end up in a mental war. Like brainwashing. One says one thing about the other. Then comes the other and says quite another thing. I do not know who I'm going to believe?!

Further difficult situations emerged when they felt they were not good enough, for instance, when told that they were overweight. However, the young persons described how their initiatives to take up sports and recreational activities were met with distrust as the parents thought the young persons were incapable of interacting socially in a proper way. The young persons did not think they had any such problems; they said they felt that they were like everybody else and thus able to fit in. Feelings of powerlessness emerged when there were no or only a few alternative solutions for the young persons. This also happened when parents and support people asked for the young person's opinions after decisions had already been made. Further sources of dissatisfaction were parents who were too controlling regarding the young person's everyday life.

#### Stress and rest

The challenge of managing the emotional span between feeling stressed and feeling rested contributed to the young persons’ vulnerability in everyday life. The feeling of stress was affected by several factors. The young persons thought that feeling they had too much to do was a significant stress factor in their everyday life. However, they also described how they sometimes had difficulties understanding and explaining the consequences of stress. More radical life changes, such as moving to a new house, were stressful if the young persons were not prepared sufficiently in advance. Everyday social situations involving a lot of people made the young persons feel unfocused and vulnerable. Obsessive thoughts about harming others were a challenge to manage and were described as raising the level of stress too high and making the situations frightening.

Carina: You know what it is like, some idiot asks you to hold someone's child. You know what absolutely must not happen and that takes up all your thoughts. One should not throw the kid on the floor, or throw it out of the window. One should not say mean things about the kid.

Carina: It is a total stress factory.

Feeling tense was described as finding it difficult to relax physically. Not being able to ignore schoolwork prevented the young persons from being fully relaxed. Being able to relax was described as doing nothing, just being, or as changing their usual state of inactivity to one of planning for leisure activities with friends.

Being tired was considered by the young persons as a starting point for feeling stressed, which, in turn, led to more tiredness and guilt about not getting things done. An ordinary school day followed by homework was perceived as very tiring. However, not having so much to do during the day was also a challenge as it made it difficult to manage the tiredness and encouraged daytime sleeping. The implementations of strategies leading to an ordinary diurnal rhythm were hampered. The young persons described how they felt drained of energy when their peers used them to listen to their problems. Being unable to stop analyzing what happened in social life was exhausting. Other reasons for being drained of energy are illustrated in the following quotation:

Coach: Fatigue, is there something special now that makes you so exhausted?

Kevin: Everything. Finding jobs, school, help at home, being with people, moving. Having too much to think about.

Problems with sleep were described as feeling tired on waking up, finding it difficult to fall asleep at bedtime and also sleeping poorly at night. Sometimes medication containing melatonin had to be used to induce sleep. Not getting to bed in time and turning night into day and vice versa resulting in oversleeping were explained as a part of the young person's personality; being a “night person.”

#### When feelings and thoughts are a concern

Vulnerability in everyday life was affected by situations when the young persons’ feelings and thoughts were a concern. For instance, thoughts about being overweight and the conflict between feeling satisfied with one's own body and knowing that a lower weight would be more healthy and socially acceptable were described and aroused feelings of ambivalence and reduced self-esteem. One young person was afraid of not being able to cope with the intrusive thoughts of nausea and the feelings of disgust which in turn caused anxiety. The young person concluded that this fear was close to an obsession. Being very obsessive was described by the young persons as having “mantras” that became more disturbing in personal emotional downturns. Shame and guilt were concerns expressed by the young persons, especially when the obsessive thoughts had connotations with sexual values. It was usually possible to control the obsessive thoughts, but not always.

Feeling anxious was described in terms of: uncertainty about changing school; the consequences of conflict resolution in class; fitting in at a new job with new demands; anxiety in the mornings; and feeling trapped in a crowd of people.

David: Anxiety in itself breeds more anxiety, when you think about how bad you sometimes feel. Later in the day it tends to get better, and I can cook and move about.

When the young persons said that they were feeling nervous, it was essentially connected with social relations and tensions in the family. Feelings of discomfort were a consequence of losing control during emotional downturns, or when parents themselves were uncontrolled and verbally abusive. Depression was described in terms of feeling sad and could occur for no special reason and for short periods, but was also said to be a sadness always there in the background. Tense situations in class were a source of sadness when self-control broke down and tears were close. The young persons’ descriptions of feeling angry were related to family problems and exemplified as being easily provoked by other people in social situations.

### Struggling to find a life of one's own

#### Decide and carry out

The focus of everyday life was on making decisions and getting things done to achieve a life of one's own. Decision-making was associated with being hindered both by the young persons themselves and by others around them.

Håkan: I feel, however, that other people often try to decide for me. A bit like I'm not regarded as an adult even though I am. Although I might not feel like one, I must still be young at heart.

The young persons described how they tried to encourage themselves by proceeding with their decisions and to change negative ways of thinking through using new strategies. They planned to take control in everyday life by the struggling to keep track of important appointments and deadlines and performing everyday duties on a regular basis; for instance, preparing meals, making weekly purchases, physical training, and a diurnal rhythm of sleeping and waking. Strategies to find a suitable education and a job were outlined by the young persons. They also decided to change the direction of their lives by, for example, not playing video games or participating in Internet games, but rather in social relations. A prerequisite for the planning to succeed was to write everything down. Carrying out less important things also had to be scheduled. Getting things done was exemplified in this quotation:

Jan: But I can often do things quite quickly and if it is something absolutely essential, then I cannot stop doing it until I have finished, but then on the other hand I cannot do other important things because “prio1” is the only thing I think about.

The young persons described how they realized that their planning was on a low level in relation to that of those who they described as “other people.” In general, poor planning had consequences for their studies; they were unprepared at the start of courses; and had insufficient money for purchasing textbooks. They described successful planning to obtain support for their studies required acknowledging their shortcomings and then deciding what support to ask for. It included taking time to ask the teachers for more specific information to ensure that they had understood instructions for homework and study assignments. Another way was to plan to manage their whole life situation on their own, but, as a precaution, to decide who to contact if something went wrong.

The young persons said that they liked to have control, which was integrated with self-discipline. Consequently, reducing control was the same as reducing self-discipline and the ability to carry out important tasks which could lead to chaos. However, too many ideas about too many solutions when trying to decide were divisive and inhibited rational choices. One strategy for achieving inner control was to decide to keep their things in order and not let too much chaos build up. Scheduling to avoid chaos by cleaning up little by little was one way of taking control over everyday life. One way to control the NPD was to create routines for taking medications. The problem of managing time was described by a young person as follows:

David: I am a time optimist, postponing most things for the future and I do it when it really needs to be done.

In their efforts to get things done the young persons said they wanted to be able to do many things simultaneously but described how they became frustrated when they tried.

#### Making life choices

The young persons’ struggle to find a life of their own involved making significant life choices starting with everyday life. The young persons wanted to succeed in life, and making demands on themselves was described as a strategy for achieving their goals, but sometimes their own requirements became a burden. Another strategy was to do their very best and thereby feel good enough.

Frida: My goal is to become an animal nurse or whatever it is called. I would never manage to become a veterinary. There are too many courses to read.

The young persons described clear ideas about their future professions. In order to find possible courses they used the Internet. They extended their ideas by looking for other kinds of work than they had before. Sometimes the job search resulted in several jobs, and then the problem was to call and decline. It could also be a good experience to be sought after, to have the expertise.

Jan: And now, a month ago, I was, offered another job I would much prefer and which I think would suit me perfectly.

#### Taking care of oneself

Taking care of oneself independently in everyday activities was for the young persons a struggle in the striving for a life of one's own. Caring for themselves included going to appointments on their own without parents, finding the way to new places, managing to take the right bus, being alone at home, and managing to get ready in the morning without reminders from parents.

Coach: … I remember we talked about the importance of having clean clothes. Do you fix it yourself or do you need to be reminded?

Gabriel: I can handle it by myself

Gabriel: I have done that for years

The young persons described how they wished to manage independently the usual chores of everyday life, such as getting to school on time and handling money. Managing meals could cause some problems as it was difficult to establish regular eating habits. To cook their own meals could also be problematic as the young persons described it was difficult of finding varied dishes for dinner. However, breakfast was no problem at all. Difficulties in managing on one's own were caused by not having enough money, which to some extent could be controlled by the parents. The young persons said they knew that their parents did not have confidence in their ability to handle their finances and were not, therefore, sufficiently supportive. The young persons described how they wanted life to be easier and to have someone with whom they could exchange ideas about life.

## Discussion

The findings show that those who participated in the study had an everyday life affected by serious, problematic situations as well as by strengths, with a wish to find adequate solutions and a role in society. The possibilities that everyday life offers a young person are related to society's norms and where the young person is situated in the transition from childhood to adulthood. It is essential to consider the areas of work, love, and world views when exploring identity (Arnett, 2000). These areas can also be seen in the data in the present study, but to a varying degree.

In the present study, the theme “fighting against an everyday life lived in vulnerability” reveals that young persons with ADHD and ASD are exposed to “difficult things” such as unfair treatment, disappointments, and rejections by friends and loved ones. Such negative experiences, according to Arnett (2000), are part of identity exploration in emerging adulthood, which emerging adults normally cope with on their own, without family and parents. However, as became visible in our study, young persons with ADHD and ASD do not always have the “tools” with which to manage these failures. This is in line with Matheson et al. (2013) who described how impairments related to ADHD resulted in chaos as the persons with ADHD did not feel sufficiently equipped to manage everyday life. Strategies are a part of executive function, with which persons with ADHD and ASD have problems (Barkley & Murphy, 2012; Nydén et al., 2010). Finding strategies relevant for the situation, and then using them are examples of “tools” that are important for managing problematic stations in everyday life. The findings also reveal experiences of difficult situations at school. Being accepted by peers, as described by Swords et al. (2011), hinges on whether or not the ADHD is thought to be caused by personal stress and therefore the sufferer's own responsibility. This should be an important topic for IBSC, as it offers the opportunity to reflect on how to tell friends about the causes and consequences of ADHD and ASD. However, considering the grim situations young persons with ADHD and ASD end up in, evidenced in the present study, a general approach targeting students’ perceptions of mental illness should be considered. Educational interventions in secondary school targeting, e.g., the life situation for persons with ADHD and ASD could be a possible approach as such interventions may have a positive impact on attitudes and myths about mental illnesses (Sakellari, Leino-Kilpi, & Kalokerinou-Anagnostopoulou, 2011; Sakellari, Sourander, Kalokerinou-Anagnostopoulou, & Leino-Kilpi, 2014). Our study shows feelings of “stress and rest” and a span between these poles to which the young persons had to relate in everyday life was part of being vulnerable. For instance, a stressful everyday life overwhelmed the young person with ADHD and ASD, leading to fatigue. It is known that high levels of the stress hormone cortisol, resulting in self-perceived stress, show a correlation with impulsiveness in adults with ADHD (Hirvikoski, Lindholm, Nordenström, Nordström, & Lajic, 2009). The connection between stress and the number of stressors and impulsiveness should be a significant topic for the IBSC, as it probably explains many of the “ad hoc solutions” and mistakes in everyday life. This knowledge could relieve the young persons of guilt and shame, and the pathophysiological explanation model could alleviate the feeling of being the source of all evil. The participants in our study described how stress caused their sleeping problems. In a study focusing on adults with ADHD (Van Veen, Kooij, Boonstra, Gordijn, & Van Someron, 2010), the majority of participants deviated from the norm regarding their diurnal rhythm, indicating that co-morbid sleeping problems are common. Children and adolescents with ASD also have problems with the onset and maintenance of sleep, and daytime sleepiness (Goldman, Richdale, Clemons, & Malow, 2012; Paavonen et al., 2008). Healthcare professionals need to be aware of these phenomena in order to give young persons with ADHD and ASD sufficient support, coupled with reasonable demands. One suggestion made by college students in the Meaux et al. (2009) study was to set alarms on mobile phones to act as reminders in a tiring and stressful everyday life. The subtheme describing “when feelings and thoughts are a concern” revealed the young persons’ negative thoughts and feelings closely related to their own person, and also their depressed mood. Feeling in a low mood, sadness and feeling anxious and angry, which was also described in our study, was almost twice as often described in adolescents with high symptoms of ADHD than in a referent group with few symptoms of ADHD (Whalen et al., 2002). Emotional difficulties in terms of negative emotions have also been described among adults with ASD (Samson, Huber, & Gross, 2012). In our study, the young persons exemplified situations that caused anxiety as difficult to master and intruding on everyday life. To improve the possibility of managing negative emotions and thoughts and reducing vulnerability, a person-centered caring approach could be used (cf. Ekman et al., 2011). Preferably this could be provided through IBSC which allows for personal narratives on an everyday life basis.

Our study indicates, as became visible in the theme “struggling to find a life of one's own,” that an independent everyday life, important for the transition into adulthood, was not common among the young persons with ADHD and ASD. The subtheme “decide and carry out” revealed that decisions were important for the young persons with ADHD and ASD, and that they struggled to think in new directions and to find new ways in which they could get things done, take control of their life, and decide for themselves. Deciding for oneself, independently of parents, and having a relationship with one's parents on equal terms were reported as being significant in exercising an adult role by a general population of young adults in studies by Arnett (1997, 2001). In our study, the young persons prepared for exercising an adult role by improving their skills in performing in everyday life.

In our study, the subtheme “making life choices” showed that the young persons were burdened by their own demands. Our results reveal that young persons with ADHD and ASD reflect on education and work and have positive expectations concerning their chances of finding a suitable job. However, a more negative picture was drawn by Cederlund et al. (2008) for young men with AS and autism, who could not find employment. Arnett (2000) points out the benefits young people derive from trying several jobs as they simultaneously develop their adult identity while exploring various work alternatives. Because of the young persons’ experiences of living with a disability the limitations may become more evident than the possibilities regarding getting and managing a job (Billstedt et al., 2005; Cederlund et al., 2008; Kessler et al., 2006). Perhaps the transition to the adult role of a working citizen is therefore already inhibited in adolescence. If true, this would restrict the opportunities of young persons with ADHD and ASD to achieve a broad life experience, including meeting people in new situations, which is important for expanding the area of worldviews in “emerging adulthood” (Arnett, 2000). The subtheme “taking care of oneself” revealed that some of the young persons with ADHD and ASD described their parents as too controlling which could make the young person frustrated. Parental efforts to give sufficient support may instead sometimes further complicate everyday life (Waite & Ramsey, 2010) and thus, in a longer term, complicate the transition into adulthood. This can be a dilemma as young persons with ADHD and ASD need support, as was evident in the present study. However, families who have a member with ASD worry (Graetz, 2010) and, perhaps because of the lack of sufficient guidance from the healthcare system, parents are unaware of how they can support the young person. Such situations should spur healthcare professionals to find ways to support the family adequately, preferably as a whole unit. A family unit approach to caring, with the possibility of inviting all family members, could be a good starting point and make it possible to explore the need of the family as a whole and to provide adequate support (Benzein, Hagberg, & Savemann, 2008; Wright & Leahy, 1999). The young persons with ADHD and ASD strove to take care of themselves, to the very best of their ability, to meet the expectations they perceived their parents and society had of them as “emerging adults.” Perhaps the expectations that previous generations had of youths—to take on the role of an adult at 18 years of age, having finished their education, to get a job, and have a family (Arnett, 2000), are still the most prevalent, and force these young persons with ADHD and ASD into a premature and stressful adult role. Instead, parents, school staff, and healthcare professionals should support them in order to provide a less stressful transition period into adulthood, through encouraging, e.g., an extended school attendance, gaining access to and participation in trainee schemes, a long preparation for independent living, and accepting a personal assistant who could serve as a role model.

One limitation of the present study is the small sample; only 12 persons participated and not all completed the IBSC, which resulted in less written data than expected. On the other hand, in this study the data analysed comprised only a part of the total chat-log text. This indicates that the young persons made serious efforts to express themselves as they chatted with the coaches, discussing everyday life and events. The differences in psychological, physiological, and social development in persons from 15 to 26 years may be considered as a weakness of the study. Adolescents and young adults with ADHD and/or ASD are difficult to reach in terms of clinical monitoring and support. To offer an Internet-based intervention was an attempt to reach these young persons and facilitate their contact with healthcare. Content analysis according to Krippendorff (2004) is well suited to chat-log analysis. The authors’ different perspectives deepened the analysis, and credibility was established (Leininger, 1994) with each step of the analytical process being scrutinized and reflected on in detailed discussions between the two authors.

It is not possible to generalize findings from a qualitative study such as this. However, transferring relevant parts of the findings (Leininger, 1994; Patton, 2002) to other young persons with ADHD and ASD will hopefully mediate a more profound understanding of their everyday life and make their situation more visible.

## Conclusion

This study described how young persons with ADHD and ASD function and how they manage their everyday life: fighting against an everyday life lived in vulnerability and a struggle to find a life of one's own. This study revealed that young persons with ADHD and ASD may have a laborious existence and a need for support throughout their emerging adulthood. The clinical implications are that it is important that healthcare professionals acknowledge the situation of everyday life in their contact with young persons with ADHD and ASD, and provide long-term support and a caring relation. This study led to more in-depth knowledge about these young persons’ everyday lives through data obtained as a result of support and coaching over the Internet. This showed their ability to use IBSC to express the complexity of everyday life for young persons with ADHD and ASD. Using e-coaching in this population makes available new opportunities for healthcare professionals to acknowledge these young persons’ problems, and that online coaching can facilitate the contact between the young persons and the medical and healthcare services.
