# Corrigendum

**DOI:** 10.3402/qhw.v9.25425

**Published:** 2014-07-11

Regarding the paper titled: Difficulties in everyday life: “Young persons with attention-deficit/hyperactivity disorder and autism spectrum disorders perspectives. A chat-log analysis” *by Britt H. Ahlström and Elisabet Wentz*

Published in *International Journal of Qualitative Studies on Health and Well-being*, 28 May 2014. Citation: Int J Qualitative Stud Health Well-being 2014, **9**: 23376 - http://dx.doi.org/10.3402/qhw.v9.23376

On page 3, first paragraph (“*Internet-based support and coaching”*), the last sentence currently reads: “The coaches chatted with the participants from their work places or homes.”

This sentence should read: “The coaches chatted with the participants from their work places or homes **(Wentz, Nydén & Krevers, 2012)**.”

The references have been supplemented with: “Wentz, E., Nydén., A. & Krevers, B. (2012). Development of an internet-based support and coaching model for adolescents and young adults with ADHD and autism spectrum disorders: A pilot study. *European Child and Adolescent Psychiatry, 21*(11), 611–622. doi: 10.1007/s00787-012-0297-2.”

